# Reading the glyco-code: New approaches to studying protein–carbohydrate interactions

**DOI:** 10.1016/j.sbi.2022.102395

**Published:** 2022-05-30

**Authors:** Simon Wisnovsky, Carolyn R. Bertozzi

**Affiliations:** 1 Faculty of Pharmaceutical Sciences, University of British Columbia, Vancouver, BC, V6T 1Z3, Canada; 2 Department of Chemistry, Stanford University, Stanford, CA, 94305, USA; 3 Howard Hughes Medical Institute, Stanford, CA, 94305, USA

## Abstract

The surface of all living cells is decorated with carbohydrate molecules. Hundreds of functional proteins bind to these glycosylated ligands; such binding events subsequently modulate many aspects of protein and cell function. Identifying ligands for glycan-binding proteins (GBPs) is a defining challenge of glycoscience research. Here, we review recent advances that are allowing protein-carbohydrate interactions to be dissected with an unprecedented level of precision. We specifically highlight how cell-based glycan arrays and glycogenomic profiling are being used to define the structural determinants of glycan-protein interactions in living cells. Going forward, these methods create exciting new opportunities for the study of glycans in physiology and disease.

## Introduction

**Figure 1:**
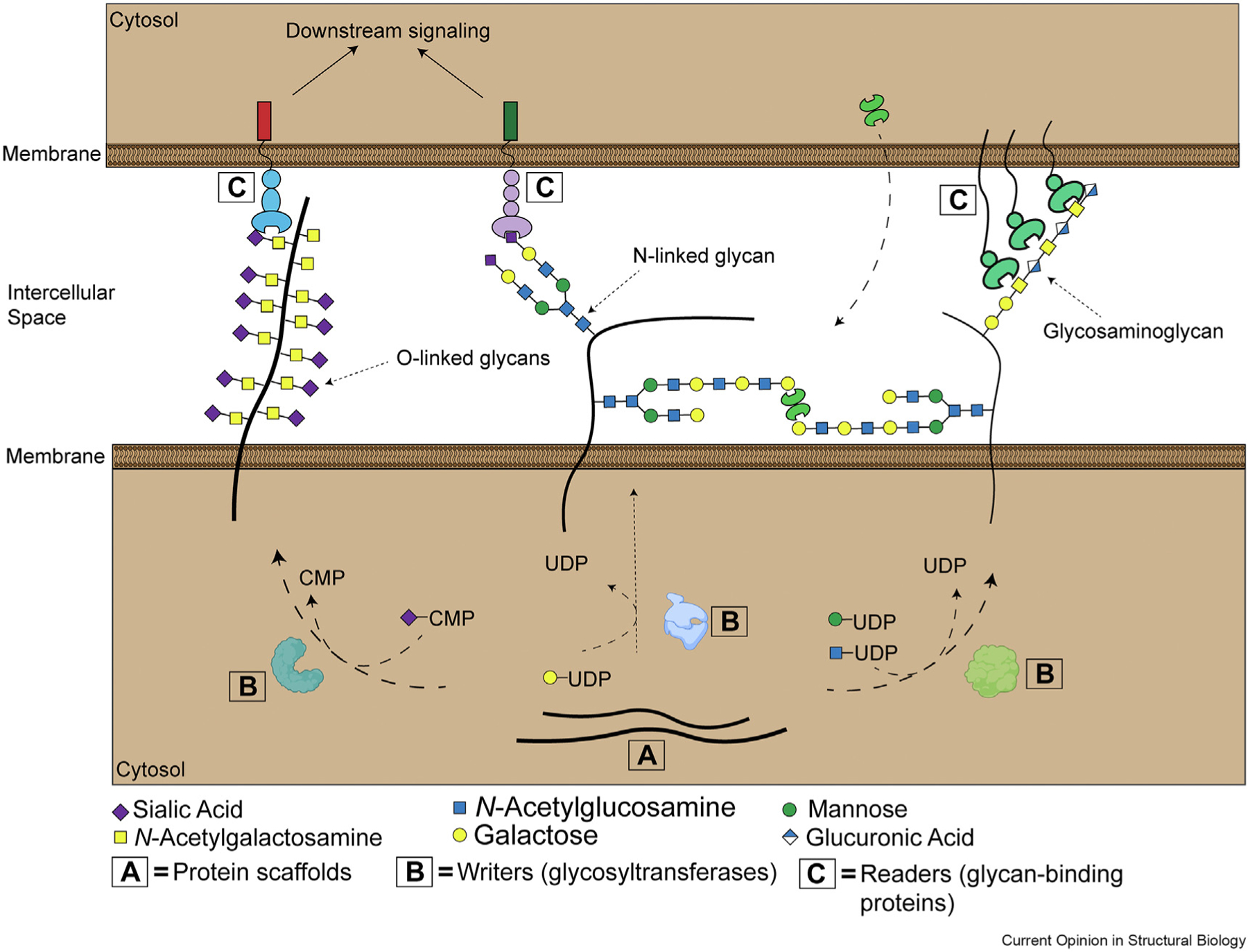
The cell surface glyco-code. *Scaffolds* are the proteins or lipids to which carbohydrate chains are attached. These molecules play a key role in the presentation of glycans on the cell surface. *Writers* are enzymes (principally glycosyltransferases) that build complex carbohydrate chains. These enzymes can directly link a glycan to a protein/lipid. They can also extend glycans by covalently linking new monosaccharide building blocks to existing oligosaccharide chains. *Readers* are glycan-binding proteins that bind to specific glycan-containing molecular motifs, which are termed *ligands*. Often, binding between a reader and its ligand will initiate transduction of downstream biological signaling pathways. UDP = Uridine diphosphate, CMP = Cytidine-5-monophosphate.

Cellular glycosylation is a molecular language of staggering complexity. The “letters” of the human glycocode consist of 10 monosaccharides that can linked together in many different ways [1]. Resulting cell surface *glycans* are produced through the combined activity of hundreds of *writer* enzymes, principally glycosyltransferases. Writer enzymes build oligosaccharide chains that are covalently attached to thousands of protein and lipid *scaffolds* [1]. The resulting collection of glycoprotein and glycolipid antigens encode biological information that is subsequently translated by *glycan-binding proteins (GBPs)*. These factors can be thought of as *readers* of the cellular glyco-code. Binding between a reader protein and a glycosylated ligand can trigger cell signalling pathways, drive cell–cell adhesion and nucleate new protein–protein interactions (Figure 1) [1]. Recent advances in translational glycoscience (reviewed in Ref. [2]) have underlined the significance of these protein-glycan interactions to the pathogenesis and treatment of disease. For example, the *Siglec* family of glycan-binding immune receptors have become hot targets for cancer immunotherapy in recent years [3]. Siglecs are inhibitory receptors that bind to sialic acid-containing glycan ligands frequently overexpressed on the surface of cancer cells [3]. Blockade of Siglec signalling with targeted antibodies, enzymatic removal of cell surface sialoglycans, and targeted suppression of sialic acid synthesis have all recently been shown to induce potent antitumour immune responses *in vivo* [4–6]. Other GBPs, such as the soluble *galectins* and the functionally diverse *C-type lectin* family, are similarly emerging as targets for various diseases [7–10].

Every GBP possesses at least one cognate *ligand* that regulates its biological function. Characterization of these ligands can produce key insights into the physiological role of a specific GBP. Ligands, once identified, can also provide new molecular targets for therapeutic intervention [4,11]. Identification of ligands for GBPs, however, is not a simple task. Glycan-protein interactions are influenced by a combination of several factors. Firstly, glycans vary both in their monosaccharide composition and in the stereochemistry of the linkages between sugar subunits. Most GBPs exhibit both monosaccharide and linkage-based binding preferences [1,3]. Secondly, while some GBPs display little specificity beyond recognition of the sugar itself, many other GBPs can interact with non-carbohydrate elements of protein and lipid scaffolds in ways that strengthen both the affinity and specificity of binding [12,13]. Finally, glycan-protein interactions are frequently influenced by the valency and topology of glycan presentation on the cell surface. Prior work has convincingly demonstrated that a GBP can show markedly different affinities for the same glycan structure depending on how closely glycan units are clustered together on the cell membrane [14,15].

As a result of this complexity, it is first important to clearly define what constitutes a ligand for a GBP. In our view, ligands should not always be thought of as isolated glycan structures that can be attached to many redundant scaffolds. Numerous studies have demonstrated that the nature of the underlying scaffold can be critical for understanding glycan-protein binding events [12,16]. It is also inadequate merely to identify glycosylated proteins that happen to be bound by a GBP. Many proteins exist in multiple glycosylation states, and often only one of these glycoforms will actually serve as a functional ligand [17–19]. It is instead best to conceptualize a ligand as a 3-dimensional macromolecular motif that exhibits a distinguishing set of carbohydrate, proteinaceous and topological features. How do we define what these features are for any given ligand? In this Mini-Review, we highlight recent methodological advances that are providing new options for researchers interested in this question.

## Affinity capture methods for mapping protein–glycan interactions

One classical approach to defining ligands for GBPs is affinity capture of interacting proteins from cell or tissue lysates using recombinant GBPs as bait molecules. Analytical techniques like mass spectrometry can then be used to identify and characterize the glycosylation states of protein interactors. While these methods are as old as glycoscience itself, technical advances in mass spectrometry-based glycoproteomics continue to improve their sensitivity and resolution [20]. In recent years, for example, this basic strategy has been applied to identify novel interactors of immune-regulatory GBPs [21–24] and microbial glycoproteases [25], among many other examples. New platforms for engineering soluble, multimeric GBPs with higher target-binding affinities also provide improved tools for conducting these studies [26].

In some cases, glycan-protein interactions cannot be preserved under the denaturing conditions required for cell lysis and affinity capture. A number of key studies have shown that such transient glycan-protein contacts can be effectively captured through the use of proximity labeling approaches [27–31]. In one version of this strategy, recombinant GBPs are genetically fused to an enzyme like ascorbate peroxidase (APEX) or horseradish peroxidase (HRP) [27,29]. These reagents are then incubated with intact cells. An enzyme-catalyzed reaction is subsequently performed that covalently biotinylates proteins in close proximity to the GBP. Affinity capture and mass spectrometry can then be used to identify novel scaffolding proteins that might interact with the GBP [27–31]. Notably, proximity labeling methods vary significantly in the range (the “diffusion radius”) within which interacting proteins are labelled. Different methods can thus be expected to capture different interacting partners, which may bind the GBP either directly or indirectly [27–31].

**Figure 2:**
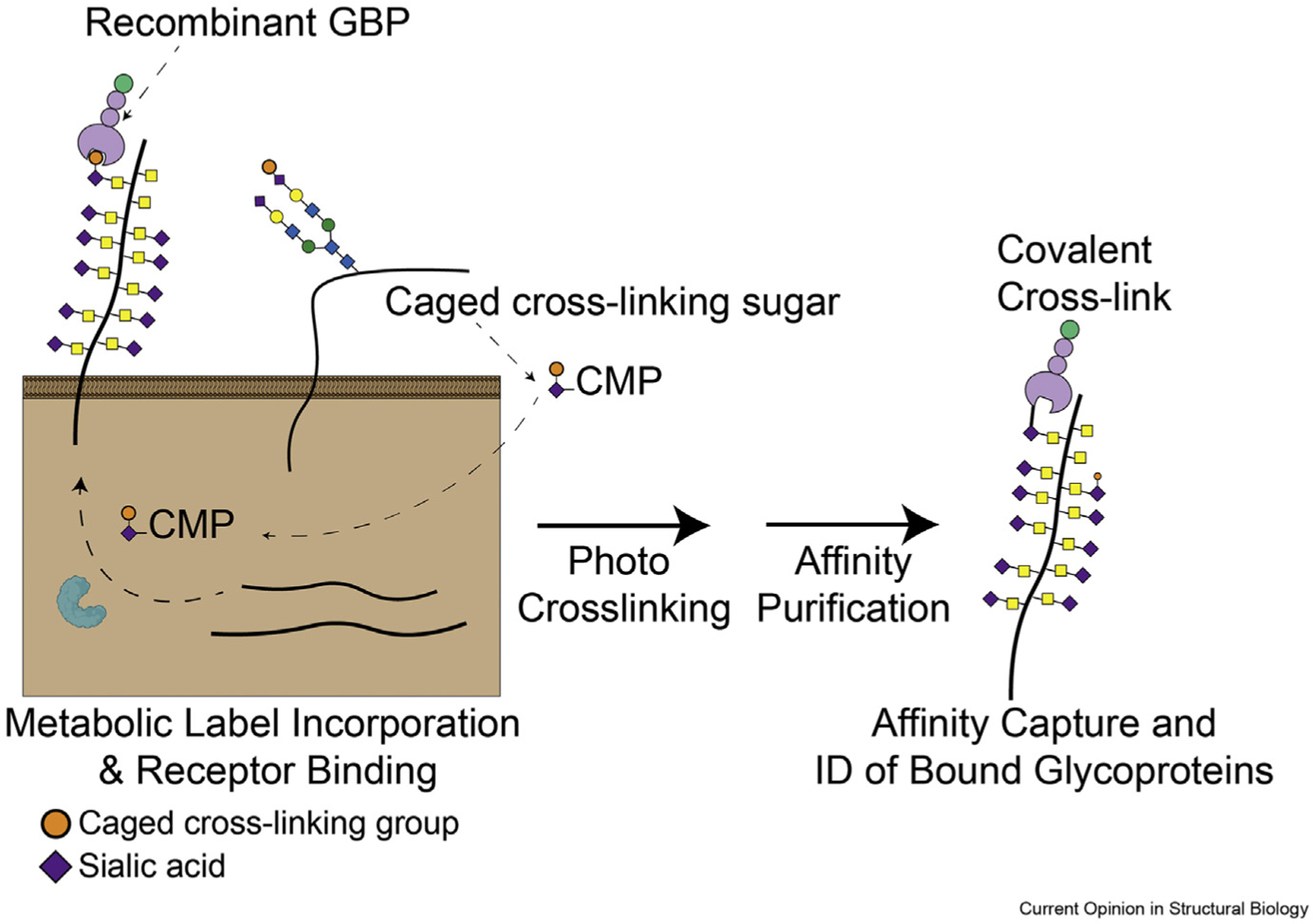
Affinity capture of GBP-binding ligands. In one approach, cells are incubated with a synthetic monosaccharide modified with a photocrosslinking group. After incorporation of this sugar into cell surface glycans, cells are incubated with a recombinant GBP. Exposure of cells to light induces the formation of covalent cross-links between the GBP and its ligands. Proteins can then be affinity purified and subjected to glyocoproteomic analysis to identify glycoproteins bound by the GBP.

Another notable advance has been the development of photocrosslinking monosaccharide analogues, which can be used to chemically fix glycan-protein interactions. In this approach, intact cells are incubated with a synthetic monosaccharide bearing a caged cross-linking group, which is subsequently incorporated into a broad range of cell surface glycans (Figure 2). Cells can then be incubated with recombinant GBPs, which will selectively bind to a subset of these glycans. Following exposure to light, the cross-linking moiety reacts to form covalent links between the GBP and any glycoprotein scaffolds to which the GBP has bound. Simple affinity purification protocols can then be used to identify these cross-linked scaffolding proteins and their appended glycans. These techniques were recently well-reviewed in Ref. [32].

Photocrosslinking sialic acid analogues represent one class of tools that have been developed and used to study glycan-protein interactions in recent years [33–35]. More recently, the applicability of this technique has been greatly expanded by the generation of the first photocrosslinking *N*-acetylglucosamine analogue, which can be used to broadly identify N-glycosylated ligands for various GBPs [36]. The development of these tools has been paralleled by the arrival of new methods for engineering cellular glycosylation pathways. Recent reports have shown how “bump-hole” enzyme engineering strategies can expand the scope of chemically modified sugars accepted by the cellular glycan biosynthetic machinery, further expanding possible applications of future photocrosslinkers [37–40]. Methods development in this space has been rapid, and it will be exciting to see whether these techniques can be integrated and applied to a wider range of biological questions. It is not possible, however, to profile glycan-protein binding through biochemical approaches alone. In pulldown experiments, for example, cell lysis removes proteins from their native cell membrane context, which can be a crucial factor influencing GBP-ligand interactions. Proteins that interact with recombinant GBPs in a pulldown experiment will not necessarily act as functional ligands for the GBP *in situ*. More specialized approaches to studying these binding events in intact cells are thus also required.

## The future of glycan arrays

Unlike proteins, which can be easily recombinantly expressed, glycans must either be chemically synthesized or isolated from natural sources [41]. If enough distinct glycans can be accessed and purified, these molecules can be coupled to a solid support, generating a synthetic “glycan microarray” [41]. These arrays can then be probed with recombinant GBPs, yielding basic insights into oligosaccharide-binding specificity [41]. Recent leaps in the automation and accessibility of high-throughput glycan synthesis have greatly expanded the range of glycan structures that can be interrogated using this method [42,43]. While synthetic glycan arrays are now a mature technology (see Ref. [41] for a more comprehensive review), these tools continue to generate important findings. One recent study, for example, used glycan arrays to discover that the co-stimulatory immune receptor CD28 binds *in vitro* to glycans containing sialic acid. This initial finding sparked further biological investigation that revealed a whole new role for sialoglycans in regulation of T-cell activation [44]. Additionally, a huge amount of glycan array data is now easily accessible to the glycobiology community. These results can thus be leveraged to generate new discoveries through the application of modern data analysis pipelines. One recent work, for example, systematically analyzed a range of publicly available glycan microarray results with a novel machine learning algorithm [45]. This approach correctly predicted several aspects of GBP binding specificities that had been missed by prior studies [45].

**Figure 3:**
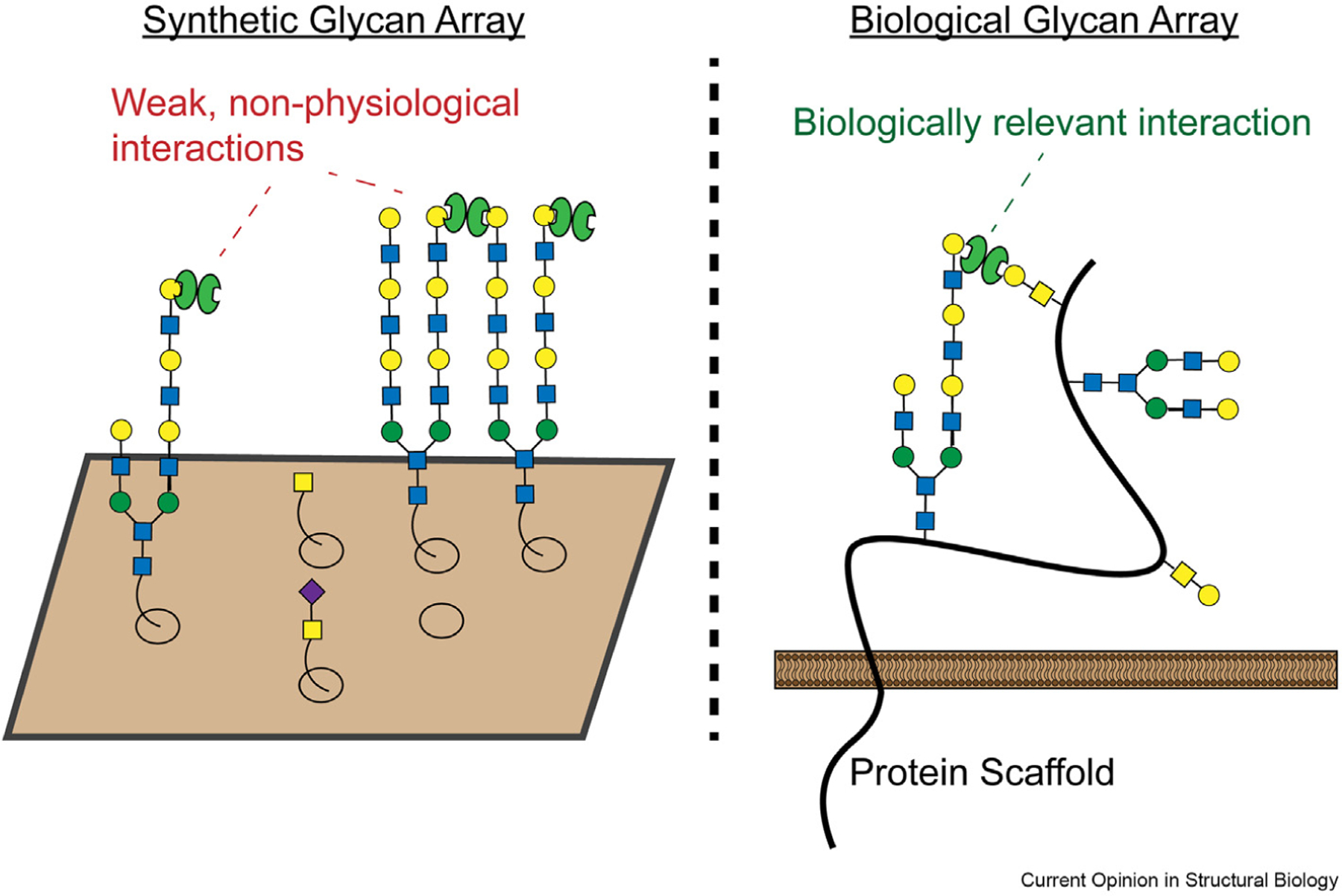
Synthetic and biological glycan arrays. Synthetic arrays may contain glycans that form components of a physiological ligand for a GBP, but these glycans may not be presented in the correct valency and orientation to mediate high-affinity binding events. Synthetic glycans may also be anchored to a solid support via flexible linkers that don’t recapitulate physiological interactions with a protein scaffold. Engineering of cell surface glycans by genetic or chemical approaches, conversely, allows for dissection of glycan-binding interactions in their correct biochemical context.

Synthetic glycan arrays have some important limitations, however. Glycans are typically attached to an inert solid support via a non-natural linker; biologically relevant factors like glycan valency and clustering are not well recapitulated (Figure 3) [41]. There has thus long been interest in developing new chemical approaches (such as the synthesis of bio-mimetic glycopolymers) that allow glycans to be presented in a more biologically relevant context [46,47]. Several recent papers have reported new breakthroughs in this area, using a variety of innovative approaches.

In one study, researchers used Chinese hamster ovary (CHO) cells as a platform to construct “cell-based glycan arrays” [48]. As these cells express a limited repertoire of glycan structures on their surface, *in vitro* treatment with a small panel of recombinant glycosyl-transferases allowed for installation of fucose and sialic acid into cell surface glycans in a linkage-specific manner [48]. The investigators successfully used this platform to identify a high-affinity ligand for the immune receptor Siglec-15 [48]. Another study recently reported the coupling of synthetic glycans to the surface of bacteriophage particles [49]. These efforts yielded a “liquid glycan array”: a library of glycosylated phages where every glycan structure was associated with a specific DNA barcode. Crucially, this platform allows for tunable control of the valency with which glycans are displayed on the virus capsid. Pulldown of phage particles with GBPs, followed by deep sequencing of barcoded phage libraries, revealed optimal glycan structures and densities required to enable protein-glycan interactions [49]. In future, encoding glycan structural information into easily sequenced DNA libraries will significantly augment the ease and sensitivity of glycan array experiments [49].

CRISPR-Cas9 genome engineering can also be used to edit glycan biosynthesis in ways that allow dissection of GBP binding specificities [50–52]. In one recent study, researchers used CRISPR-Cas9 gene editing to generate a panel of HEK293 cell lines bearing knockouts and knock-ins of key glycosyltransferase enzymes. This effort produced a glycosylation “atlas”: a collection of cell lines exhibiting a wide range of cell surface glycosylation patterns [50,51]. In a subsequent work, these glyco-engineered cell lines were transfected with a panel of O-glycoprotein reporter constructs. Binding of transfected cells to recombinant Siglecs was then assessed, allowing for systematic dissection of both glycan and scaffolding protein-binding specificities. Intriguingly, the authors found that some Siglecs exhibited specificity for certain O-glycoprotein reporters. Additionally, these specificities changed upon perturbation of O-glycan biosynthesis [53]. This effort highlighted the complex interplay between carbohydrate and protein structural elements in determining Siglec ligand affinities. In another study, researchers used the same technology to profile the carbohydrate binding and cleavage specificity of glycoprotein-specific proteases, demonstrating the wide applicability of this approach [54]. Going forward, these biological glycan arrays are ripe for application towards studying a huge range of GBPs that have not been fully characterized using *in vitro* approaches.

## Applying functional genomics to discover novel ligands for GBPs

**Figure 4:**
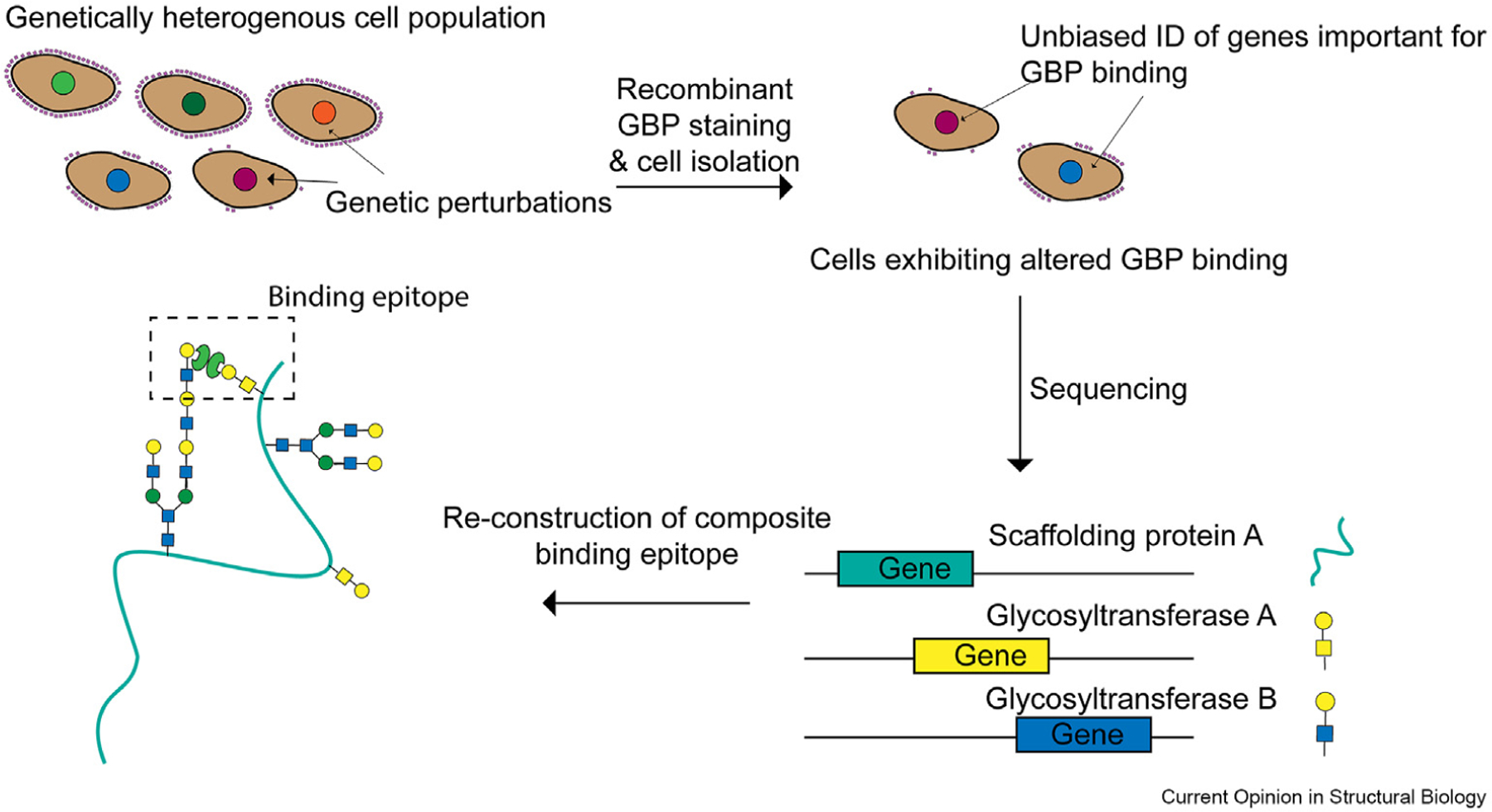
Glyco-genomic profiling of GBP-ligand interactions. A genetically heterogenous cell population is either generated through CRISPR genome-wide mutagenesis or isolated from physiological sources. Cells exhibiting altered (either diminished or elevated) binding to a glycan-binding protein are isolated (via FACS) and genetically characterized by high-throughput sequencing. This method allows unbiased identification of genes required for expression of ligands for the given glycan-binding protein, information that can be used to infer structural characteristics of the ligand.

Expression of a ligand for a GBP can require co-expression and repression of many genes (glycosyltransferases, glycosidases and scaffolding proteins) within a discrete biosynthetic circuit. Recent work has highlighted how cutting-edge functional genomics screening methods can be applied towards mapping these pathways (Figure 4). In one recent study, researchers transduced Cas9-expressing cells with a genome-wide library of short guide RNAs (sgRNAs) and isolated cells (via FACS) that showed reduced binding to recombinant Siglecs. sgRNAs were then sequenced to identify genes whose knockdown reduces expression of glycan ligands for these GBPs [55]. This screening study identified a set of glycosyltransferases that synthesize a specific glycan (the disialyl core 1 tetrasaccharide), as well as a specific O-linked glycoprotein called CD43. Extensive biochemical characterization revealed that Siglec-7 binds to a specific epitope at the N-terminus of CD43, which is decorated with clusters of adjacent disialyl core 1 residues [55]. Both the structure of the glycan and the nature of the scaffold are critical for enabling binding, exemplifying how composite glycopeptide motifs often act as ligands for GBPs.

Functional genomics can also be used to characterize the upstream factors that regulate these biosynthetic pathways. One work recently used CRISPR screening to show that the transcription factor KDM2B regulates ligand expression for heparan sulfate-binding proteins by coordinating expression of multiple sulfotransferase enzymes [56]. A related study used bioinformatic analysis to identify ZNF263 as another key transcription factor directing heparan sulfate biosynthesis [57]. Coupling of RNA-seq with lectin microarray technology has revealed that miRNAs act as master regulators of glycan biosynthetic pathways ([58] recently well-reviewed in Ref. [59]). Finally, recent ground-breaking studies have applied scRNA-seq to correlate specific transcriptomic states with glycan structure and abundance in both primary Tcells [60] and human stem cells [61]. Taken together, these strategies provide new avenues for profiling the genetic circuits that underlie distinct cellular “glyco-states”, with exciting future implications for characterizing the drivers of glycanprotein interactions.

## Conclusions & future perspectives

CRISPR functional genomics and single cell profiling methods will continue to become more closely integrated with glycoscience in future years. In particular, we expect newer CRISPR-based technologies, such as programmable epigenetic silencing and base editing, to generate further opportunities at the genomics/glycomics interface [62,63]. It is also notable that attempts to study the glycome with functional genetics have so far used only single gene knockout methods. Cellular glycostates, however, frequently emerge from the concerted activity of many functionally redundant genes [1]. High-throughput screening methods that allow multiplexed, simultaneous disruption of several genes in combination are ideally suited to the study of these complex glycostates. The continuous refinement of such techniques [64,65] will enable the study of glycan-protein interactions in future.

The closely timed development of cell-based glycan arrays and glyco-genomic profiling methods has been another major theme in recent years. There are some parallels between these approaches. Recent application of these two techniques to discovery of Siglec ligands, for example, has produced some strikingly similar (although not identical) findings [53,55]. These strategies are not redundant, however, but complementary. Genome-wide screening studies cannot achieve the precise control over cellular glycosylation that is possible with targeted genetic or chemical engineering. On the other hand, the ability to broadly assess the function of thousands of genes in a single experiment, rather than a more limited array of glycosyltransferases and scaffolds, may uncover unexpected biology that would be missed by array-based studies. Interestingly, resources are now being developed that may offer a productive synthesis of these two approaches. In one recent work, for example, investigators constructed a pooled sgRNA library targeting all known glycosyltransferase enzymes and applied this library in high-throughput screens to rapidly identify genetic determinants of Selectin binding to living cells [66]. We anticipate these types of glycobiology-specific screening tools will continue to be developed and will be of great use to the research community.

Lastly, we highlight that new research is challenging fundamental assumptions in glycoscience. It has long been thought that glycans can only be attached to protein and lipid scaffolds. Genetic approaches to studying glycosylation have historically rested on the assumption that ligands for GBPs will be generated by the protein-coding genome. The recent discovery of “glyco-RNA” conjugates, and the related finding that these glycosylated nucleic acids may bind to certain Siglecs, has turned this assumption on its head [67]. These studies remind us that much remains to be discovered about how glycans regulate macromolecular interactions, and hint at the exciting work that remains to be done.

## References

[1] Varki A, Cummings RD, Esko JD, Stanley P, Hart GW, Aebi M, Darvill AG, Kinoshita T, Packer NH, Prestegard JH, : In Essentials of glycobiology. Cold Spring Harbor Laboratory Press; 2015. Copyright 2015–2017 by The Consortium of Glycobiology Editors, Jolla La, California. All rights reserved. 27010055

[2] Smith BAH, Bertozzi CR: The clinical impact of glycobiology: targeting selectins, Siglecs and mammalian glycans. Nat Rev Drug Discov 2021, 20:217–243. 33462432 10.1038/s41573-020-00093-1 PMC7812346

[3] Duan S, Paulson JC: Siglecs as immune cell checkpoints in disease. Annu Rev Immunol 2020, 38:365–395. 31986070 10.1146/annurev-immunol-102419-035900

[4] Barkal AA, Brewer RE, Markovic M, Kowarsky M, Barkal SA, Zaro BW, Krishnan V, Hatakeyama J, Dorigo O, Barkal LJ, : CD24 signalling through macrophage Siglec-10 is a target for cancer immunotherapy. Nature 2019, 572:392–396. 31367043 10.1038/s41586-019-1456-0 PMC6697206

[5] Wang J, Sun J, Liu LN, Flies DB, Nie X, Toki M, Zhang J, Song C, Zarr M, Zhou X, : Siglec-15 as an immune suppressor and potential target for normalization cancer immunotherapy. Nat Med 2019, 25:656–666. 30833750 10.1038/s41591-019-0374-x PMC7175920

[6] Gray MA, Stanczak MA, Mantuano NR, Xiao H, Pijnenborg JFA, Malaker SA, Miller CL, Weidenbacher PA, Tanzo JT, Ahn G, : Targeted glycan degradation potentiates the anticancer immune response in vivo. Nat Chem Biol 2020, 16:1376–1384. 32807964 10.1038/s41589-020-0622-x PMC7727925

[7] Dusoswa SA, Verhoeff J, Abels E, Méndez-Huergo SP, Croci DO, Kuijper LH, de Miguel E, Wouters V, Best MG, Rodriguez E, : Glioblastomas exploit truncated O-linked glycans for local and distant immune modulation via the macrophage galactose-type lectin. Proc Natl Acad Sci Unit States Am: USA 2020, 117:3693–3703. 10.1073/pnas.1907921117 PMC7035608 32019882

[8] Yang R, Sun L, Li C-F, Wang Y-H, Yao J, Li H, Yan M, Chang W-C, Hsu J-M, Cha J-H, : Galectin-9 interacts with PD-1 and TIM-3 to regulate T cell death and is a target for cancer immunotherapy. Nat Commun 2021, 12:832. 33547304 10.1038/s41467-021-21099-2 PMC7864927

[9] Lujan AL, Croci DO, Rabinovich GA, Damiani MT: Galectins as potential therapeutic targets in STIs in the female genital tract. Nat Rev Urol 2022, 19:240–252. 35105978 10.1038/s41585-021-00562-1

[10] Thiemann S, Baum LG: Galectins and immune responses-just how do they do those things they do? Annu Rev Immunol 2016, 34:243–264. 26907217 10.1146/annurev-immunol-041015-055402

[11] Krishnamurthy VR, Sardar MY, Ying Y, Song X, Haller C, Dai E, Wang X, Hanjaya-Putra D, Sun L, Morikis V, : Glycopeptide analogues of PSGL-1 inhibit P-selectin in vitro and in vivo. Nat Commun 2015, 6:6387. 25824568 10.1038/ncomms7387 PMC4423566

[12] Sako D, Comess KM, Barone KM, Camphausen RT, Cumming DA, Shaw GD: A sulfated peptide segment at the amino terminus of PSGL-1 is critical for P-selectin binding. Cell 1995, 83:323–331. 7585949 10.1016/0092-8674(95)90173-6

[13] Hashimoto N, Ito S, Tsuchida A, Bhuiyan RH, Okajima T, Yamamoto A, Furukawa K, Ohmi Y, Furukawa K: The ceramide moiety of disialoganglioside (GD3) is essential for GD3 recognition by the sialic acid-binding lectin SIGLEC7 on the cell surface. J Biol Chem 2019, 294:10833–10845. 31138648 10.1074/jbc.RA118.007083 PMC6635455

[14] Cohen M, Varki A: Modulation of glycan recognition by clustered saccharide patches. Int Rev Cell and Mol Biol 2014, 308: 75–125. 24411170 10.1016/B978-0-12-800097-7.00003-8

[15] Hakomori Si SI: The glycosynapse. Proc Natl Acad Sci U S A 2002, 99:225–232. 11773621 10.1073/pnas.012540899 PMC117543

[16] Marcelo F, Supekar N, Corzana F, van der Horst JC, Vuist IM, Live D, Boons G-JPH, Smith DF, van Vliet SJ: Identification of a secondary binding site in human macrophage galactose-type lectin by microarray studies: implications for the molecular recognition of its ligands. J Biol Chem 2018, 294:1300–1311. 30504228 10.1074/jbc.RA118.004957 PMC6349122

[17] Velazquez F, Grodecki-Pena A, Knapp A, Salvador AM, Nevers T, Croce K, Alcaide P: CD43 functions as an E-selectin ligand for Th17 cells in vitro and is required for rolling on the vascular endothelium and Th17 cell recruitment during inflammation in vivo. J Immunol 2016, 196:1305–1316. 26700769 10.4049/jimmunol.1501171 PMC4724552

[18] Sackstein R, Merzaban JS, Cain DW, Dagia NM, Spencer JA, Lin CP, Wohlgemuth R: Ex vivo glycan engineering of CD44 programs human multipotent mesenchymal stromal cell trafficking to bone. Nat Med 2008, 14:181–187. 18193058 10.1038/nm1703

[19] Dimitroff CJ, Lee JY, Fuhlbrigge RC, Sackstein R: A distinct glycoform of CD44 is an L-selectin ligand on human hematopoietic cells. Proc Natl Acad Sci Unit States Am 2000, 97: 13841. 10.1073/pnas.250484797 PMC17663 11095749

[20] Riley NM, Bertozzi CR, Pitteri SJ: A pragmatic guide to enrichment strategies for mass spectrometry-based glycoproteomics. Mol Cell Proteomics 2020, 20:100029. 33583771 10.1074/mcp.R120.002277 PMC8724846

[21] Pirro M, Schoof E, van Vliet SJ, Rombouts Y, Stella A, de Ru A, Mohammed Y, Wuhrer M, van Veelen PA, Hensbergen PJ: Glycoproteomic analysis of MGL-binding proteins on acute T-cell leukemia cells. J Proteome Res 2019, 18:1125–1132. 30582698 10.1021/acs.jproteome.8b00796 PMC6399673

[22] Yoshimura A, Asahina Y, Chang L-Y, Angata T, Tanaka H, Kitajima K, Sato C: Identification and functional characterization of a Siglec-7 counter-receptor on K562 cells. J Biol Chem 2021, 296:100477. 33640457 10.1016/j.jbc.2021.100477 PMC8040268

[23] Gonzalez-Gil A, Li TA, Porell RN, Fernandes SM, Tarbox HE, Lee HS, Aoki K, Tiemeyer M, Kim J, Schnaar RL: Isolation, identification, and characterization of the human airway ligand for the eosinophil and mast cell immunoinhibitory receptor Siglec-8. J Allergy Clin Immunol 2021, 147: 1442–1452. 32791164 10.1016/j.jaci.2020.08.001 PMC7876160

[24] Gonzalez-Gil A, Porell RN, Fernandes SM, Wei Y, Yu H, Carroll DJ, McBride R, Paulson JC, Tiemeyer M, Aoki K, : Sialylated keratan sulfate proteoglycans are Siglec-8 ligands in human airways. Glycobiology 2018, 28:786–801. 29924315 10.1093/glycob/cwy057 PMC6142871

[25] Malaker SA, Riley NM, Shon DJ, Pedram K, Krishnan V, Dorigo O, Bertozzi CR: Revealing the human mucinome. bio-Rxiv 2021, 10.1101/2021.01.27.428510:2021.2001.2027.428510. PMC9209528 35725833

[26] Rodrigues E, Jung J, Park H, Loo C, Soukhtehzari S, Kitova EN, Mozaneh F, Daskhan G, Schmidt EN, Aghanya V, : A versatile soluble siglec scaffold for sensitive and quantitative detection of glycan ligands. Nat Commun 2020, 11:5091. 33037195 10.1038/s41467-020-18907-6 PMC7547722

[27] Joeh E, O’Leary T, Li W, Hawkins R, Hung JR, Parker CG, Huang ML: Mapping glycan-mediated galectin-3 interactions by live cell proximity labeling. Proc Natl Acad Sci Unit States Am 2020, 117:27329. 10.1073/pnas.2009206117 PMC7959530 33067390

[28] Vilen Z, Joeh E, Critcher M, Parker CG, Huang ML: Proximity tagging identifies the glycan-mediated glycoprotein interactors of galectin-1 in muscle stem cells. ACS Chem Biol 2021, 16:1994–2003. 34181849 10.1021/acschembio.1c00313 PMC8922946

[29] Chang L, Chen YJ, Fan CY, Tang CJ, Chen YH, Low PY, Ventura A, Lin CC, Chen YJ, Angata T: Identification of siglec ligands using a proximity labeling method. J Proteome Res 2017, 16:3929–3941. 28899088 10.1021/acs.jproteome.7b00625

[30] Xie Y, Sheng Y, Li Q, Ju S, Reyes J, Lebrilla CB: Determination of the glycoprotein specificity of lectins on cell membranes through oxidative proteomics. Chem Sci 2020, 11:9501–9512. 34094216 10.1039/d0sc04199h PMC8162070

[31] Wu G, Nagala M, Crocker PR: Identification of lectin counter-receptors on cell membranes by proximity labeling. Glycobiology 2017, 27:800–805. 28810661 10.1093/glycob/cwx063 PMC5881670

[32] Wu H, Kohler J: Photocrosslinking probes for capture of carbohydrate interactions. Curr Opin Chem Biol 2019, 53: 173–182. 31706134 10.1016/j.cbpa.2019.09.002 PMC7192017

[33] McCombs JE, Zou C, Parker RB, Cairo CW, Kohler JJ: Enhanced cross-linking of diazirine-modified sialylated glycoproteins enabled through profiling of sialidase specificities. ACS Chem Biol 2016, 11:185–192. 26541974 10.1021/acschembio.5b00775 PMC4731091

[34] Bond MR, Whitman CM, Kohler JJ: Metabolically incorporated photocrosslinking sialic acid covalently captures a ganglioside-protein complex. Mol Biosyst 2010, 6: 1796–1799. 20625600 10.1039/c0mb00069h PMC2953467

[35] Han S, Collins BE, Bengtson P, Paulson JC: Homomultimeric complexes of CD22 in B cells revealed by protein-glycan cross-linking. Nat Chem Biol 2005, 1:93–97. 16408005 10.1038/nchembio713

[36.*] Wu H, Shajahan A, Yang JY, Capota E, Wands AM, Arthur CM, Stowell SR, Moremen KW, Azadi P, Kohler JJ: A photo-cross-linking GlcNAc analog enables covalent capture of N-linked glycoprotein-binding partners on the cell surface. Cell Chemical Biology 2021, 29:84–97. 34331854 10.1016/j.chembiol.2021.07.007 PMC8792112

[37] Schumann B, Malaker SA, Wisnovsky SP, Debets MF, Agbay AJ, Fernandez D, Wagner LJS, Lin L, Li Z, Choi J, : Bump-and- Hole engineering identifies specific substrates of glycosyltransferases in living cells. Mol Cell 2020, 78:824–834. e815. 32325029 10.1016/j.molcel.2020.03.030 PMC7276986

[38] Cioce A, Bineva-Todd G, Agbay AJ, Choi J, Wood TM, Debets MF, Browne WM, Douglas HL, Roustan C, Tastan OY, : Optimization of metabolic oligosaccharide engineering with Ac(4)GalNAlk and Ac(4)GlcNAlk by an engineered pyrophosphorylase. ACS Chem Biol 2021, 16:1961–1967. 33835779 10.1021/acschembio.1c00034 PMC8501146

[39] Cioce A, Calle B, Marchesi A, Bineva-Todd G, Flynn H, Li Z, Tastan OY, Roustan C, Keenan T, Both P, : Cell-specific bioorthogonal tagging of glycoproteins in Co-culture. bioRxiv 2021, 10.1101/2021.07.28.454135:2021.2007.2028.454135.

[40] Cioce A, Malaker SA, Schumann B: Generating orthogonal glycosyltransferase and nucleotide sugar pairs as next-generation glycobiology tools. Curr Opin Chem Biol 2021, 60: 66–78. 33125942 10.1016/j.cbpa.2020.09.001 PMC7955280

[41] Gao C, Wei M, McKitrick TR, McQuillan AM, Heimburg-Molinaro J, Cummings RD: Glycan microarrays as chemical tools for identifying glycan recognition by immune proteins. Front Chem 2019, 7. 10.3389/fchem.2019.00833 PMC6923789 31921763

[42] Joseph AA, Pardo-Vargas A, Seeberger PH: Total synthesis of polysaccharides by automated glycan assembly. J Am Chem Soc 2020, 142:8561–8564. 32338884 10.1021/jacs.0c00751 PMC7304863

[43] Li T, Liu L, Wei N, Yang J-Y, Chapla DG, Moremen KW, Boons G-J: An automated platform for the enzyme-mediated assembly of complex oligosaccharides. Nat Chem 2019, 11:229–236. 30792508 10.1038/s41557-019-0219-8 PMC6399472

[44.*] Edgar LJ, Thompson AJ, Vartabedian VF, Kikuchi C, Woehl JL, Teijaro JR, Paulson JC: Sialic acid ligands of CD28 suppress costimulation of T cells. ACS Cent Sci 2021, 7:1508–1515. 34584952 10.1021/acscentsci.1c00525 PMC8461770

[45] Bojar D, Meche L, Meng G, Eng W, Smith DF, Cummings RD, Mahal LK: A useful guide to lectin binding: machine-learning directed annotation of 57 unique lectin specificities. ACS Chem Biol 2022, 10.1021/acschembio.1c00689. PMC9679999 35084820

[46] Huang ML, Cohen M, Fisher CJ, Schooley RT, Gagneux P, Godula K: Determination of receptor specificities for whole influenza viruses using multivalent glycan arrays. Chem Commun 2015, 51:5326–5329. 10.1039/c4cc08613a PMC4359031 25574528

[47] Rabuka D, Forstner MB, Groves JT, Bertozzi CR: Noncovalent cell surface engineering: incorporation of bioactive synthetic glycopolymers into cellular membranes. J Am Chem Soc 2008, 130:5947–5953. 18402449 10.1021/ja710644g PMC2724873

[48.*] Briard JG, Jiang H, Moremen KW, Macauley MS, Wu P: Cell-based glycan arrays for probing glycan–glycan binding protein interactions. Nat Commun 2018, 9:880. 29491407 10.1038/s41467-018-03245-5 PMC5830402

[49.**] Sojitra M, Sarkar S, Maghera J, Rodrigues E, Carpenter EJ, ** Seth S, Ferrer Vinals D, Bennett NJ, Reddy R, Khalil A, : Genetically encoded multivalent liquid glycan array displayed on M13 bacteriophage. Nat Chem Biol 2021, 17: 806–816. 33958792 10.1038/s41589-021-00788-5 PMC8380037

[50] Narimatsu Y, Joshi HJ, Nason R, Van Coillie J, Karlsson R, Sun L, Ye Z, Chen Y-H, Schjoldager KT, Steentoft C, : An atlas of human glycosylation pathways enables display of the human glycome by gene engineered cells. Mol Cell 2019, 75:394–407. e395. 31227230 10.1016/j.molcel.2019.05.017 PMC6660356

[51] Büll C, Joshi HJ, Clausen H, Narimatsu Y: Cell-based glycan arrays-A practical guide to dissect the human glycome. STAR Protocols 2020, 1:100017. 33111073 10.1016/j.xpro.2020.100017 PMC7580198

[52] Stolfa G, Mondal N, Zhu Y, Yu X, Buffone A, Neelamegham S: Using CRISPR-Cas9 to quantify the contributions of O-glycans, N-glycans and Glycosphingolipids to human leukocyte-endothelium adhesion. Sci Rep 2016, 6:30392. 27458028 10.1038/srep30392 PMC4960646

[53] Büll C, Nason R, Sun L, Van Coillie J, Madriz Sørensen D, Moons SJ, Yang Z, Arbitman S, Fernandes SM, Furukawa S, : Probing the binding specificities of human Siglecs by cell-based glycan arrays. Proc Natl Acad Sci Unit States Am: USA 2021, 118:e70506, 10.1073/pnas.2026102118. PMC8092401 33893239

[54] Nason R, Büll C, Konstantinidi A, Sun L, Ye Z, Halim A, Du W, Sørensen DM, Durbesson F, Furukawa S, : Display of the human mucinome with defined O-glycans by gene engineered cells. Nat Commun 2021, 12:4070. 34210959 10.1038/s41467-021-24366-4 PMC8249670

[55.**] Wisnovsky S, Möckl L, Malaker SA, Pedram K, Hess GT, Riley NM, Gray MA, Smith BAH, Bassik MC, Moerner WE, : Genome-wide CRISPR screens reveal a specific ligand for the glycan-binding immune checkpoint receptor Siglec-7. Proc Natl Acad Sci Unit States Am: USA 2021, 55:118. 10.1073/pnas.2015024118 PMC7865165 33495350

[56.*] Weiss RJ, Spahn PN, Chiang AWT, Liu Q, Li J, Hamill KM, Rother S, Clausen TM, Hoeksema MA, Timm BM, : Genome-wide screens uncover KDM2B as a modifier of protein binding to heparan sulfate. Nat Chem Biol 2021, 17: 684–692. 33846619 10.1038/s41589-021-00776-9 PMC8159865

[57] Weiss RJ, Spahn PN, Toledo AG, Chiang AWT, Kellman BP, Li J, Benner C, Glass CK, Gordts P, Lewis NE, : ZNF263 is a transcriptional regulator of heparin and heparan sulfate biosynthesis. Proc Natl Acad Sci Unit States Am: USA 2020, 117:9311–9317. 10.1073/pnas.1920880117 PMC7196839 32277030

[58] Agrawal P, Kurcon T, Pilobello KT, Rakus JF, Koppolu S, Liu Z, Batista BS, Eng WS, Hsu K-L, Liang Y, : Mapping post-transcriptional regulation of the human glycome uncovers microRNA defining the glycocode. Proc. Natl. Acad. Sci. U.S.A 2014, 111:4338–4343. 24591635 10.1073/pnas.1321524111 PMC3964104

[59] Thu CT, Mahal LK, Sweet Control: MicroRNA regulation of the glycome. Biochemistry 2020, 59:3098–3110. 31585501 10.1021/acs.biochem.9b00784 PMC10018745

[60.**] Kearney CJ, Vervoort SJ, Ramsbottom KM, Todorovski I, Lelliott EJ, Zethoven M, Pijpers L, Martin BP, Semple T, Martelotto L, : SUGAR-seq enables simultaneous detection of glycans, epitopes, and the transcriptome in single cells. Sci Adv 2021, 7:eabe3610, 10.1126/sciadv.abe3610. 33608275 PMC7895430

[61] Minoshima F, Ozaki H, Odaka H, Tateno H: Integrated analysis of glycan and RNA in single cells. iScience 2021, 24:102882, 10.1016/j.isci.2021.102882. 34401666 PMC8349903

[62] Anzalone AV, Randolph PB, Davis JR, Sousa AA, Koblan LW, Levy JM, Chen PJ, Wilson C, Newby GA, Raguram A, : Search-and-replace genome editing without double-strand breaks or donor DNA. Nature 2019, 576:149–157. 31634902 10.1038/s41586-019-1711-4 PMC6907074

[63] Nuñez JK, Chen J, Pommier GC, Cogan JZ, Replogle JM, Adriaens C, Ramadoss GN, Shi Q, Hung KL, Samelson AJ, : Genome-wide programmable transcriptional memory by CRISPR-based epigenome editing. Cell 2021, 184:2503–2519. e2517. 33838111 10.1016/j.cell.2021.03.025 PMC8376083

[64] Wong AS, Choi GC, Cui CH, Pregernig G, Milani P, Adam M, Perli SD, Kazer SW, Gaillard A, Hermann M, : Multiplexed barcoded CRISPR-Cas9 screening enabled by CombiGEM. Proc Natl Acad Sci Unit States Am: USA 2016, 113:2544–2549. 10.1073/pnas.1517883113 PMC4780610 26864203

[65] Reis AC, Halper SM, Vezeau GE, Cetnar DP, Hossain A, Clauer PR, Salis HM: Simultaneous repression of multiple bacterial genes using nonrepetitive extra-long sgRNA arrays. Nat Biotechnol 2019, 37:1294–1301. 31591552 10.1038/s41587-019-0286-9

[66] Zhu Y, Groth T, Kelkar A, Zhou Y, Neelamegham S: A GlycoGene CRISPR-Cas9 lentiviral library to study lectin binding and human glycan biosynthesis pathways. Glycobiology 2021, 31:173–180. 32776087 10.1093/glycob/cwaa074 PMC8022984

[67.**] Flynn RA, Pedram K, Malaker SA, Batista PJ, Smith BAH, Johnson AG, George BM, Majzoub K, Villalta PW, Carette JE, : Small RNAs are modified with N-glycans and displayed on the surface of living cells. Cell 2021, 184:3109–3124. e3122. 34004145 10.1016/j.cell.2021.04.023 PMC9097497

